# Correlations between SIRT1 gene polymorphisms and diabetic kidney disease

**DOI:** 10.1098/rsos.171871

**Published:** 2018-06-13

**Authors:** Xin-Ge Yue, Zai-Gang Yang, Yue Zhang, Gui-Jun Qin, Fei Liu

**Affiliations:** 1 Department of Geriatric Endocrinology, First Affiliated Hospital of Zhengzhou University, Zhengzhou, Henan, People's Republic of China; 2 Department of Endocrinology, First Affiliated Hospital of Zhengzhou University, Zhengzhou, Henan, People's Republic of China

**Keywords:** SIRT1, diabetic kidney disease, gene

## Abstract

To investigate the correlations between SIRT1 gene polymorphisms and diabetic kidney disease (DKD). There were 150 patients with DKD in the observation group (urinary albumin excretion rate (UAER) ≥ 300 mg 24 h^−1^), and 160 patients with a more than 10 year history of type 2 diabetes but without retinopathy and DKD (UAER < 30 mg 24 h^−1^) in the control group. Genotypes of three tagged single-nucleotide polymorphism loci (rs3818292, rs4746720 and rs10823108) in the SIRT1 gene in the two groups were detected. Risks of DKD for patients with the GG and GG + AG genotype in the rs10823108 locus of the SIRT1 gene were 2.96 and 2.92 times higher than that for AA genotype carriers, respectively. The risk of DKD for patients with the GG genotype in the rs3818292 locus was 0.23 times and 0.21 times higher than that for AA and for AA + AG genotype carriers, respectively, and the risk of DKD for patients with allele G was 0.66 times higher than that for allele A carriers. There was no significant difference in genotype frequency of rs4746720 locus gene polymorphisms between the observation and control groups. The SIRT1 gene is a genetic susceptibility gene of DKD. Mutation genotype GG and GG + AG in the rs10823108 locus can increase the risk of DKD. Mutation genotype GG and allele G in the rs3818292 locus can decrease the risk of DKD.

## 1. Introduction

Diabetic kidney disease (DKD) is one of the most severe microvascular complications of diabetes mellitus, which even happens to patients who have good long-term glycaemic control. About 20%–40% of diabetic patients develop DKD, and DKD is the primary cause of end-stage renal disease (ESRD) [1,2]. Type I and II diabetes can cause kidney disease, and even lead to ESRD. [3] The pathogenesis of DKD is concealed, and DKD can lead to irreversible end-stage renal failure, which severely affects the life expectancy and quality of life of diabetics [4]. Early diagnosis of DKD and timely intervention therapy can effectively delay or even prevent the development of ESRD. However, the pathogenesis of DKD is not yet fully understood, and previous studies have suggested that glucose, lipid metabolism disorders, oxidative stress, inflammatory reactions and multiple cytokines are closely related to the onset of DKD [5,6]. Familial aggregation of DKD and its inconsistency with blood glucose control indicate that genetic factors play an important role in the occurrence and development of DKD [7]. SIRT1 (silent mating type information regulation 2 homologue 1), a kind of NAD+ histone deacetylase [8], can regulate the function of histone and most non-histone transcription factors by deacetylation, and be involved in the regulation of many biological functions in the body, such as apoptosis, mitochondrial synthesis, inflammation, glucolipid metabolism, autophagy and anti-stress [9]. This study was to investigate the correlations between SIRT1 gene polymorphisms and DKD.

## 2. Material and methods

### 2.1. Materials

One hundred and fifty DKD patients admitted to our hospital from June 2014 to June 2016 were selected in the observation group (urinary albumin excretion rate (UAER) ≥ 300 mg 24 h^−1^), with 78 males and 72 females, of age 57.98 ± 11.41 years. One hundred and sixty diabetes mellitus patients admitted to our hospital at the same time were selected in the control group (patients without retinopathy and DKD (UAER < 30 mg 24 h^−1^)), with 88 males and 72 females, of age 58.64 ± 12.06 years. The following patients were excluded: (i) patients with DKD caused by chronic renal disease; (ii) patients with bilateral renal artery stenosis (greater than 75%); (iii) patients with NYHA classification of grade IV; (iv) patients with acute renal injury or failure within the first 3 months of the treatment; and (v) patients admitted to hospital because of stroke, short temporary ischaemic attack, acute coronary artery syndrome and heart failure within 3 months.

### 2.2. Methods

#### 2.2.1. DNA extraction

Fasting venous blood (5 ml) was extracted in the morning into EDTA-2 K anticoagulation tubes and then mixed. After centrifuging, the blood without plasma was moved into an autoclave centrifuge tube. Then DNA was extracted by the phenol–chloroform method. The DNA sediment was moved into 1.5 ml epoxy resin (EP) tubes and centrifuged at 12 000 revolutions min^−1^ for 10 min. The centrifugal radius was 13.5 cm. Then the supernatant was moved out and the DNA sediment was dried naturally. Dried DNA sediment was dissolved completely with sterile water. The DNA concentration was tested with an Eppendorf ultraviolet spectrophotometer and standardized to 50 mg l^−1^, and kept as a standby in a refrigerator at 4°C.

#### 2.2.2. SIRT1 gene polymorphism detection

Single-nucleotide polymorphism (SNP) data of the SIRT1 gene of the Chinese population were obtained through the HapMap database (www.hapmap.org) [10], and the data were imported into the HAploview software [11]. The linkage disequilibrium module was defined with 95% confidence intervals. The selection criteria of tagged SNPs included the Chinese Han population, candidate gene, the increase of the 1 kb region on gene upstream and downstream, r2 tangent point of 0.8 and less allele frequency greater than 0.1. Three tagged SNPs, rs3818292 (A > G), rs4746720 (C > T) and rs10823108 (A > G), were selected from the SIRT1 gene.

rs3818292, rs4746720 and rs10823108 loci of the SIRT1 gene were searched from the GenBank database (https://www.ncbi.nlm.nih.gov/genbank/) [12], and primers of the three loci were designed by the Primer v. 5.0 software. The PCR-RFLP method was used. Primer sequences of the rs3818292 locus: upstream primer 5'-GACAATTCCAGCCATCTCTTCT-3′, downstream primer 5'-CTCACGCCTGTAATCCTAG-3′. Primer sequences of the rs4746720 locus: upstream primer 5'-AGGGAACACAGCTAATCTAGACCA-3′, downstream primer 5'-AAAGTAAAGACAACCGAGTGCT-3′. Primer sequences of the rs10823108 locus: upstream primer 5'-TCTCAGCCTCCCAAGTAG-3′, downstream primer 5'-AGTAGTAACCAGCAGCAGTCATC-3′.

The Taqman-MGB probe was used to detect the genotype of the polymorphism site in this study. Fluorescent probes were added to the PCR reaction system. The fluorophore in the PCR reaction system was cut by 5′–3′ exonuclease (Tap enzyme) of DNA and detected with real-time fluorescence quantitative PCR. The total reaction system of PCR was 25 µl including DNA 1 µl, Taqman PCR enzyme 12.5 µl, deionized water 10.25 µl, Taqman FAM/VIC probes 1.25 µl, and upstream and downstream primers. The amplification was performed on a PCR instrument. Amplification conditions were 40 circles at 95°C for 10 s and then at 60°C for 1 min. PCR amplification products were subjected to agarose gel electrophoresis 12 g l^−1^ and ethidium bromide staining. The result of PCR amplification was observed under ultraviolet radiation.

#### 2.2.3. Sequencing verification

From the observation group and the control group, 15 patients were randomly selected three times, respectively (10% of the subjects). DNA was extracted, and primers and probes were used for PCR amplification. Amplification conditions were sent to Takala, Guangzhou for sequencing to verify the accuracy of the genotype by the Taqman method in this study. The results showed that the accuracy of the TaqMan detection method in this study was as high as 100%.

#### 2.2.4. Biochemical detection

Fasting venous blood (3 ml) was extracted from every patient in the morning, which was centrifuged for 10 min (centrifugal radius of 13.5 cm, 3000 revolutions min^−1^). Then the serum in the 1.5 ml EP tube was cryopreserved as a standby at –80°C. Haemoglobin A1C (HbA1C) detection was made by high-performance liquid chromatography. Triglyceride (TG), total cholesterol (TC), high-density lipoprotein cholesterol (HDL) and low-density lipoprotein cholesterol (LDL) were determined by the enzymic method.

### 2.3. Statistical analysis

The SPSS 21.0 software was used to analyse the data. Measurement data were given as $\bar{x}\pm \text{s}$. Colony representativeness of the sample was confirmed by an independent sample *T*-test and the Hardy–Weinberg equilibrium. Genotype frequency and allele frequency in the two groups were analysed by *χ*^2^ test. Multiple-factor analysis was made by non-conditional logistic regression analysis. There was a significant difference at *p *< 0.05.

## 3. Result

### 3.1. Basic data

The average ages in two groups were 57.98 ± 11.41 and 58.64 ± 12.06, respectively, and the average weights were 55.14 ± 12.34 and 56.27 ± 11.30, respectively. There was no significant difference in the course of diabetes (*p *= 0.758), family history (*p *= 0.569), smoking history (*p *= 0.905), high-fat diet (*p *= 0.758) and dessert consumption (*p *= 0.917) between the two groups. The results of statistical analysis showed no significant difference in age (*p *= 0.719), gender (*p *= 0.758) and weight (*p *= 0.513) in both the groups.

HbA1C (*p *< 0.001), TC (*p *= 0.005), LDL (*p *= 0.005) and body mass index (BMI) (*p *= 0.01) in the observation group were notably higher than those in the control group (*p *< 0.05). There was no significant difference in the levels of TG and HDL of serum between the two groups (*p *> 0.05) (table 1).

**Table 1.** General data of patients in both groups. (There was a significant difference at *p *< 0.05. HbA1C, Haemoglobin A1C; TG, triglyceride; HDL, high-density lipoprotein cholesterol; LDL, low-density lipoprotein cholesterol; BMI, body mass index.)

term	observation group (150)	control group (160)	*p*
age	57.98 ± 11.41	58.64 ± 12.06	0.719
gender			
male	78	88	0.758
female	72	72	
weight	55.14 ± 12.34	56.27 ± 11.30	0.513
course of diabetes	11.21 ± 7.34	12.01 ± 8.23	0.758
family history [*n* (%)]	37 (24.67%)	33 (20.63%)	0.569
smoking history (day^−1^)	7.5 ± 3.5	8.0 ± 2.5	0.905
high-fat diet (number)	57 (38%)	55 (34.38%)	0.758
dessert consumption (number)	33 (22%)	36 (22.5%)	0.917
blood pressure	122 ± 10.54	119 ± 9.86	0.514
urinary protein excretion (24 h)	2.83 ± 1.50	2.79 ± 1.38	0.672
HbA1C (%)	8.99 ± 4.13	6.66 ± 4.15	<0.001
TC (mmol l^−1^)	5.07 ± 2.25	4.21 ± 1.13	0.005
TG (mmol l^−1^)	1.94 ± 1.69	1.79 ± 1.83	0.36
HDL (mmol l^−1^)	1.15 ± 0.47	1.1 ± 0.34	0.18
LDL (mmol l^−1^)	3.23 ± 1.74	2.54 ± 0.89	0.005
BMI (kg m^−2^)	27.4 ± 3.50	25.8 ± 3.90	0.01

### 3.2. Electrophoresis results of SIRT1 gene loci polymorphism

**Figure 1.**
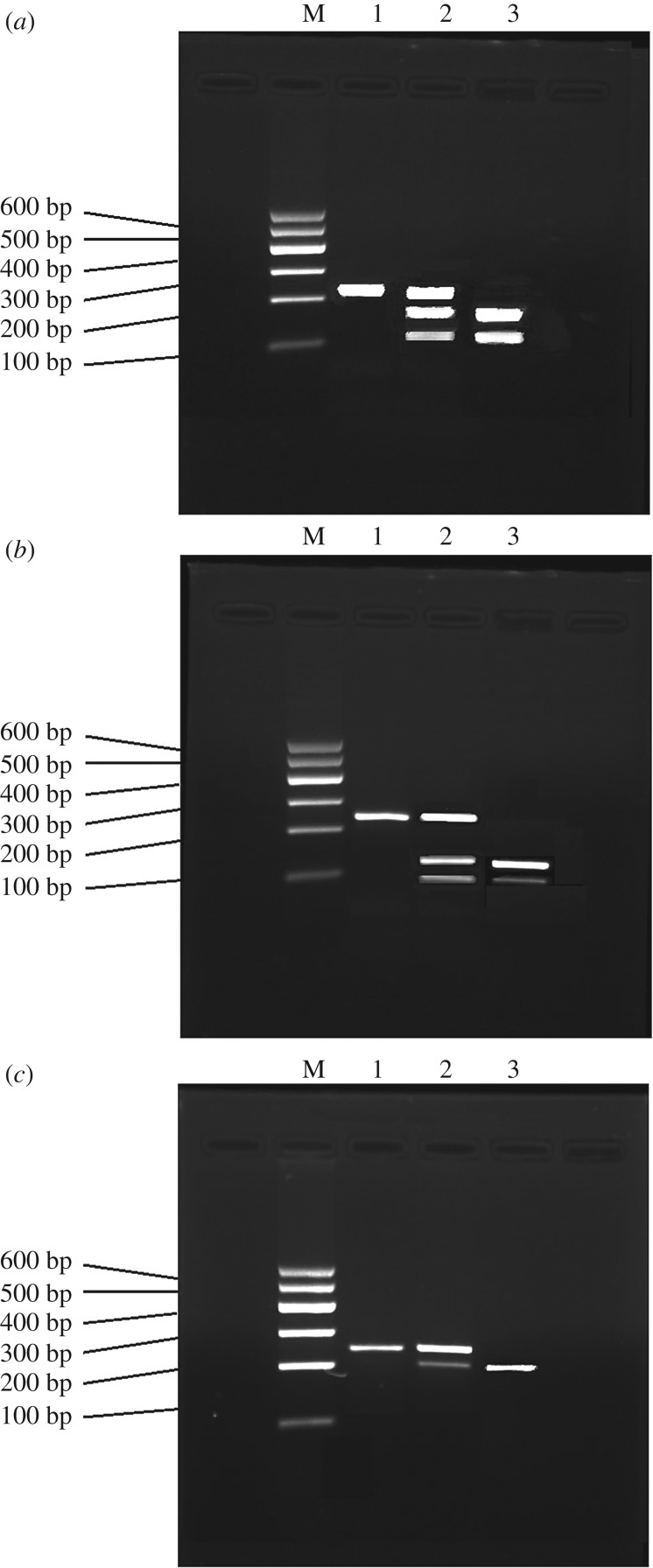
Agarose gel electrophoresis results of SIRT1 gene loci. (*a*) Electrophoresis pattern of the polymorphism genotype in the rs3818292 locus of the SIRT1 gene. (*b*) Electrophoresis pattern of the polymorphism genotype in the rs4746720 locus of the SIRT1 gene. (*c*) Electrophoresis pattern of the polymorphism genotype in the rs10823108 locus of the SIRT1 gene. M denotes marker. 1, 2 and 3 indicate GG genotype, AG genotype and AA genotype, respectively.

After the primer amplification in SIRT1 gene loci, the amplification products were subjected to agarose gel electrophoresis. The amplification results are shown in figure 1.

### 3.3. Distribution of SIRT1 loci genotype frequency in two groups

There was no significant difference in the distribution of the SIRT1 loci genotype frequency in two groups (*p *> 0.05). The distribution was consistent with the Hardy–Weinberg equilibrium [13] (table 2).

**Table 2.** SIRT1 loci genotype frequency of patients in both groups. (There was a significant difference at *p *< 0.05.)

locus		observation group (*n*, %)	control group (*n*, %)	*p*
rs3818292	AA	92 (61.3)	88 (55.0)	0.57
	GA	53 (35.4)	54 (33.8)	
	GG	5 (3.3)	18 (11.2)	
rs4746720	CC	38 (25.3)	24 (15.0)	0.93
	CT	67 (44.7)	76 (47.5)	
	TT	45 (30.0)	60 (37.5)	
rs10823108	AA	8 (5.3)	28 (17.5)	0.76
	GA	52 (34.7)	38 (23.8)	
	GG	90 (60.0)	94 (58.7)	

### 3.4. Comparison of the SIRT1 gene between two groups

The distribution of the mutation homozygous GG genotype frequency in the rs3818292 locus of the SIRT1 gene between the two groups showed a significant difference (*p *= 0.01) compared to wild homozygous type AA, and the odd ratio (OR) (95% confidence interval (CI)) value was 0.23 (0.07–0.71). The distribution of the GG genotype frequency in both groups showed a significant difference (*p *= 0.01) compared to AA + AG, and the OR (95% CI) value was 0.21 (0.07–0.72). The distribution of the mutation allele G genotype frequency showed a significant difference (*p *= 0.03) compared to allele A, and the OR (95% CI) value was 0.66 (0.43–0.96). The distribution of the mutation homozygous GG genotype frequency in the rs10823108 locus between the two groups showed a significant difference (*p *= 0.04) compared to wild homozygous type AA, and the OR (95% CI) value was 2.96 (1.08–8.58). The distribution of the GG + AG and AA genotype frequency between the two groups showed a significant difference (*p *= 0.04), and the OR (95% CI) value was 2.92 (1.09–8.41). There was no significant difference in the distribution of the genotype frequency in the rs4746720 locus between the two groups (*p *> 0.05) (table 3).

**Table 3.** Distribution of the SIRT1 loci genotype frequency and the OR (95% CI) value. (There was a significant difference at *p *< 0.05.)

locus	OR (95% CI)	*p*
rs3818292		
AA		
AG	0.93 (0.55–1.51)	0.74
GG	0.23 (0.07–0.71)	0.01
GG/AA + AG	0.21 (0.07–0.72)	0.01
GG + AG/AA	0.75 (0.45–1.19)	0.20
G /A	0.66 (0.43–0.96)	0.03
rs4746720		
CC		
CT	0.86 (0.48–1.62)	0.69
TT	0.76 (0.41–1.49)	0.45
TT /CC + CT	0.87 (0.51–1.42)	0.54
TT + CT/CC	0.83 (0.48–1.46)	0.53
T/C	0.87 (0.63–1.22)	0.45
rs10823108		
AA		
AG	2.87 (0.95–8.75)	0.06
GG	2.96 (1.08–8.58)	0.04
GG/AA + AG	1.22 (0.74–1.94)	0.46
GG + AG/AA	2.92 (1.09–8.41)	0.04
G/A	1.32 (0.91–1.98)	0.16

### 3.5. Regression analysis of correlation factors

Non-conditional logistic regression analysis was performed with ‘with or without DKD' as the dependent variables and HbA1C, TG, TC, HDL, LDL and BMI as the independent variables. The result showed that HbA1C, TC, HDL and BMI had an influence on the occurrence of DKD, and that HDL level had a negative relation with the onset of DKD (table 4).

**Table 4.** Results of T2DM multifactor and non-conditional logistic regression analysis. (There was a significant difference at *p *< 0.05. HbA1C, haemoglobin A1C; TG, triglyceride; HDL, high-density lipoprotein cholesterol; LDL, low-density lipoprotein cholesterol; BMI, body mass index.)

independent value	*B*	Wald	*P*	OR (95% CI)
HbA1C	0.06	13.78	0.000	1.06 (1.03–1.10)
TC	0.08	4.77	0.03	1.08 (1.01–1.16)
TG	−0.02	0.28	0.6	0.98 (0.91–1.06)
HDL	−0.39	6.84	0.01	0.68 (0.51–0.91)
LDL	0.09	3.36	0.007	1.09 (0.99–1.21)
BMI	2.127	3.931	0.032	1.123 (1.08–1.52)

## 4. Discussion

DKD has the feature of familial aggregation, and the genetic factor plays an important role in the pathogenesis of DKD. The regulation of histone modification has a certain influence on the DKD-related key epigenetic mechanism [14]. SIRT1, a kind of NAD+ histone deacetylase in mammals, can regulate many biological functions in the body [15]. Recently, the function of SIRT1 in the occurrence and development of renal disease has been of increasing concern in humans. SIRT1 makes a contribution towards innate cell apoptosis reduction in the kidney, kidney failure retardation caused by increase in age, inflammation reduction, renal interstitial fibrosis inhibition, blood pressure regulation, autophagy induction and protection in DKD [16–18]. In addition, SIRT1 can regulate energy metabolism under the conditions of caloric restriction and fasting through deacetylation of histones, nuclear transcription factor and related enzymes. The interdiction of SIRT1 activation can affect the development of age- and obesity-related diseases, such as diabetes, angiocardiopathy and neurodegenerative diseases [19]. The SIRT1 pathway is a new therapeutic target for DKD. Some research work has shown that SIRT1-related SNPs rs10823108, rs3818292 and rs4746720 and the level of urine protein are associated with ESRD [20]. The correlation between mutated genotypes in rs10823108, rs3818292 and rs4746720 loci and DKD needs further investigation.

In this study, the PCR-RFLP method was used to detect the distribution of the SNP genotype frequency of SIRT1 in the observation group and the control group. The result showed that patients with mutations homozygous type GG in rs10823108 had a higher risk of DKD than those with wild homozygous type AA, and that GG + GA carriers had a higher risk of disease than AA carriers. The results indicated that patients with GG in the rs10823108 locus had a higher risk of proteinuria than those with AA. Patients with GG in the rs3818292 locus had a lower risk of disease than those with AA, and allele G carriers had a lower risk of disease than allele A carriers, which indicated that the mutation genotype in the rs3818292 locus was a protective genetic susceptibility factor in the development of DKD.

Multiple logistic regression analysis showed that TC, LDL, BMI and high HbA1C level were dangerous contributing factors for DKD, which accorded with the results of a previous animal-model study [21]. Hyperlipidaemia could influence fibrinolysis and the activity of the coagulation system, and generate massive amounts of lipid peroxide. The sedimentation of lipid peroxide in blood vessel endothelium damages endothelial cells, accelerating the process of arteriosclerosis, which promotes the occurrence and development of DKD. Lipid treatment could prevent the occurrence of nephropathy and prolong its development [22]. A high level of HDL is a protective factor for DKD [23]. Hirano [24] also showed that massive proteinuria had positive correlations with the LDL level in serum for patients with overt DKD, and that the HDL level in serum notably decreased for patients with DKD with kidney failure. Long-term hyperglycaemia could cause damage to blood vessel endothelium in the whole body, reducing lipoprotein lipase activity, the increase of the TG level and the decrease of HDL concentration.

This study indicates that the SIRT1 gene is an inherited susceptibility gene of DKD. Mutation genotype GG and GG + AG in the rs10823108 locus can increase the risk of DKD. Mutation genotype GG and allele G in the rs3818290 locus can reduce the risk of DKD. The conclusions need further verification for the applicability of this research to daily clinical practice.

## Data accessibility

Our data are deposited in this manuscript. Single-nucleotide polymorphism (SNP) data of the SIRT1 gene of the Chinese population were obtained through the HapMap database (www.hapmap.org) [8]. rs3818292, rs4746720 and rs10823108 loci of SIRT1 gene were searched from the GenBank database (https://www.ncbi.nlm.nih.gov/genbank/) [10].
